# Draft Genome Sequences of *Sphingobium* sp. Strain TCM1 and *Sphingomonas* sp. Strain TDK1, Haloalkyl Phosphate Flame Retardant- and Plasticizer-Degrading Bacteria

**DOI:** 10.1128/genomeA.00668-16

**Published:** 2016-07-14

**Authors:** Yoshio Kera, Katsumasa Abe, Daisuke Kasai, Masao Fukuda, Shouji Takahashi

**Affiliations:** Department of Bioengineering, Nagaoka University of Technology, Nagaoka, Niigata, Japan

## Abstract

*Sphingobium* sp. strain TCM1 and *Sphingomonas* sp. strain TDK1 are haloalkyl phosphate flame retardant- and plasticizer-degrading bacteria. We report here the draft genome sequences of these strains to provide insights into the molecular mechanism underlying their degradation ability.

## GENOME ANNOUNCEMENT

*Sphingobium* sp. strain TCM1 and *Sphingomonas* sp. strain TDK1, isolated in Niigata, Japan, are alphaproteobacterial strains that degrade various organophosphorus flame retardants and plasticizers to utilize them as a phosphorus source, including the haloalkyl phosphate flame retardants and plasticizers Tris(1,3-dichloro-2-propyl)phosphate (TDCPP) and Tris(2-chloroethyl)phosphate (TCEP) (1, 2). TDCPP and TCEP are persistent environmental contaminants suspected to have various toxic effects on animals, such as neurotoxicity, reproductive toxicity, and carcinogenicity (3). Thus, these compounds have become a serious threat to animal and human health. Effective contaminant removal techniques are required. These strains are considered to sequentially hydrolyze the three phosphoester bonds of TDCPP and TCEP using three kinds of phosphoesterases: phosphotriesterase (PTE), phosphodiesterase (PDE), and phosphomonoesterase (PME). We identified PTEs capable of degrading TDCPP and TCEP in both strains and named them haloalkylphosphorus hydrolase (HAD) (4). However, PDEs and PMEs involved in the degradation have not yet been identified.

The genomic DNAs of TCM1 and TDK1 were sequenced by paired-end sequencing with an Illumina MiSeq platform (Illumina, San Diego, CA). The 17,545,191 and 16,896,573 reads obtained for TCM1 and TDK1, respectively, were assembled using CLC Genomics Workbench version 7.0 (CLC bio, Aarhus, Denmark). Their contigs were analyzed by Rapid Annotations using Subsystems Technology (RASTtk) (5), tRNAscan-SE version 1.23 (6), and RNAmmer version 1.2 (7). The draft genome of TCM1 has 5,154,797 bp, with 64.3% G+C content and comprises 41 contigs ranging from 5,218 to 555,109 bp, with an average coverage of 607× and a *N*_50_ length 273,373 bp. The draft genome of TDK1 has 5,414,866 bp, with 66.2% G+C content and comprises 48 contigs ranging from 5,124 to 524,221 bp, with an average coverage of 544× and a *N*_50_ length of 245,827 bp.

The annotation for TCM1 revealed 5,111 predicted coding sequences (CDSs), including 3 rRNA (5S rRNA, 16S rRNA, and 23S rRNA) and 55 tRNA genes. The annotation for TDK1 revealed 5,159 CDSs, including 3 rRNA (5S rRNA, 16S rRNA, and 23S rRNA) and 48 tRNA genes. The functional comparison of the genome sequences using the SEED Frame for Comparative Genomics (http://www.theseed.org/) revealed that the closest neighbor of TCM1 and TDK1 was *Sphingopyxis alaskensis* RB2256 (SEED Viewer identification no. 317655.9), with similarity scores of 505 and 536, respectively. A functional annotation showed that strains TCM1 and TDK1 possess numerous genes, as well as the *had* gene, for phosphorus metabolism, including genes encoding a high-affinity inorganic phosphate transport complex (PstSCAB), low-affinity inorganic phosphate transporters, a two-component regulatory system (PhoBR) for regulating Pho regulon gene expression, alkaline phosphatases, pyrophosphatases, polyphosphatases, and alkylphosphonate hydrolases. This identification and determination of the gene regulation mechanism can provide the necessary insight to develop an efficient and effective degradation technique for removing TDCPP and TCEP from the environment.

### Nucleotide sequence accession numbers.

The whole-genome shotgun projects of *Sphingobium* sp. strain TCM1 and *Sphingomonas* sp. strain TDK1 have been deposited at DDBJ/EMBL/GenBank databases under the accession numbers LXVX00000000 and LXVY00000000, respectively. The versions described in this paper are their first versions.
